# An Influenza HA and M2e Based Vaccine Delivered by a Novel Attenuated *Salmonella* Mutant Protects Mice against Homologous H1N1 Infection

**DOI:** 10.3389/fmicb.2017.00872

**Published:** 2017-05-15

**Authors:** Irshad A. Hajam, John H. Lee

**Affiliations:** College of Veterinary Medicine and Bio-Safety Research Institute, Chonbuk National UniversityIksan, South Korea

**Keywords:** influenza A virus, H1N1, immune protection, M2e, *Salmonella*, T cell responses

## Abstract

Attenuated *Salmonella* strains constitute a promising technology for the development of a more efficient multivalent protein based vaccines. In this study, we constructed a novel attenuated strain of *Salmonella* for the delivery and expression of the H1N1 hemagglutinin (HA) and the conserved extracellular domain of the matrix protein 2 (M2e). We demonstrated that the constructed *Salmonella* strain exhibited efficient HA and M2e protein expressions and little cytotoxicity and pathogenicity in mice. Using BALB/c mice as the model, we showed that the mice vaccinated with a *Salmonella* strain expressing HA and M2e protein antigens, respectively, induced significant production of HA and M2e-specific serum IgG1 and IgG2a responses, and of anti-HA interferon-γ producing T cells. Furthermore, immunization with Salmonella-HA-M2e-based vaccine via different routes provided protection in 66.66% orally, 100% intramuscularly, and 100% intraperitoneally immunized mice against the homologous H1N1 virus while none of the animals survived treated with either the PBS or the *Salmonella* carrying empty expression vector. *Ex vivo* stimulated dendritic cells (DCs) with heat killed *Salmonella* expressing HA demonstrated that DCs play an important role in the elicitation of HA-specific humoral immune responses in mice. In summary, *Salmonella*-HA-M2e-based vaccine elicits efficient antigen-specific humoral and cellular immune responses, and provides significant immune protection against a highly pathogenic H1N1 influenza virus.

## Introduction

Influenza is one of the most important viral diseases in humans caused by influenza A viruses, with significant medical and economic burdens (Meltzer and Bridges, 2003). Vaccination remains the most effective method to protect the population, including both humans and animals, against influenza infections (Chen et al., 2008; Soema et al., 2015). Although currently available influenza vaccines are effective in controlling viral infections, these vaccination strategies require a large supply of specific-pathogen free (SPF) embryonated eggs and a long timeline that could be threatened during an influenza pandemic (Chen et al., 2008; Soema et al., 2015). Further, the influenza viruses have the ability to continuously evolve either gradually through antigenic drift (point mutations) or rapidly through reassortment with another divergent virus (antigenic shift) (Nicholson et al., 2003). Consequently, the immunity generated against one vaccine strain is only protective against another strain that shares antigenically related proteins. Thus to control and prevent potential outbreaks of pandemic influenza viruses (e.g., the 2009 swine-origin H1N1 virus), an effective and broadly protective vaccine that is based on relatively conserved amino acid sequences and egg-independent production would be a promising approach. Influenza A viruses are members of the *orthomyxoviridae* family, which have enveloped, segmented, single-stranded negative sense RNA genome (Jang et al., 2009). These viruses generally cause yearly epidemics and, potentially, pandemics when an influenza virus with a novel antigenically shifted hemagglutinin (HA) emerges in a population resulting in widespread infection, high morbidity, and high mortality (Hoelscher et al., 2006). The HA is the most abundant integral viral envelope protein, and currently available vaccination strategies aim at inducing the neutralizing antibody responses by targeting the globular head of the HA protein (Chen et al., 2008; Pan et al., 2010). These strategies are only effective when HA in the vaccine antigenically matches the HA of the circulating viruses. Further, because the influenza virus strain that will cause the next epidemic or potential pandemic cannot be predicted, novel and effective vaccination strategies, therefore, should be devised that will not only provide the broad spectrum of protection, but are also readily available to modifications and allow concurrent quick production of the vaccine. Similar to HA, the matrix protein 2 (M2) is an integral transmembrane protein of influenza A viruses (Deng et al., 2015). The ectodomain of M2 (M2e), 23 amino acid residues, has been remarkably conserved in all human influenza A strains, and animal experiments have demonstrated that M2e-specific antibodies can provide cross-protective immunity against infections caused by different types of influenza A subtypes (Layton et al., 2009; Kim et al., 2013; Deng et al., 2015). Although natural infections and vaccinations with currently available influenza vaccines elicit very weak M2e-specific immune responses, presenting M2e on a suitable carrier greatly improves its immunogenicity and cross-protective efficacy (De Filette et al., 2006; Layton et al., 2009). Several M2 based vaccine candidates have been attempted including flagellin-M2e conjugates (Wang B.-Z. et al., 2012), baculovirus-expressed M2e (Zebedee and Lamb, 1988) and M2e DNA constructs that potentially express M2 (Fiers et al., 1999; Mozdzanowska et al., 2003). Although effective in eliciting M2e-specific immune responses, these vaccines were not completely protective against influenza virus infections. Besides M2e, conserved HA stalk domain has also been used to induce a broad spectrum of protection against divergent influenza strains (Steel et al., 2010; Yassine et al., 2015; Joyce et al., 2016).

In this study, we exploited the *Salmonella* enterica serovar Typhimurium (*S*. Typhimurium) as delivery system for HA and M2e, and evaluated the efficacy of the system in mice. Live attenuated *Salmonella* vaccine vectors carrying recombinant influenza HA, M2e and NA antigens have been previously used (Layton et al., 2009; Van et al., 2013; Pei et al., 2015; Je Hyoung et al., 2017). These studies have demonstrated that these vaccines elicit efficient systemic and mucosal immune responses to both the foreign and the *Salmonella* derived antigens and protect animals against influenza virus infections. *Salmonella* based vaccines have certain advantages over currently available influenza vaccines. This vaccination strategy is highly cost effective and allows for a quick response to novel influenza viruses, as it circumvents the need for a constant supply of eggs. Moreover, only antigenically important proteins from the influenza viruses are used to construct the vaccine, so the candidate offers the potential to differentiate vaccinated from infected animals. Here we report the construction of an O antigen deficient *S.* Typhimurium mutant, JOL1800 strain, expressing and secreting H1N1 HA and multiple tandem copies of M2e proteins. Our results show that coadministration of *Salmonella* strains expressing-HA (Sal-HA) and M2e (Sal-M2e) proteins, respectively, induced significant production of HA and M2e-specific humoral and cell mediated immune (CMI) responses in mice, independently of the route of vaccination. We also show that *ex-vivo* loaded DCs with heat killed Sal-HA elicited HA-specific humoral immune responses in mice. A lethal H1N1 (Influenza A Puerto Rico/8/34) virus challenge experiment shows that Sal-HA plus Sal-M2e vaccination can protect the mice from a highly pathogenic H1N1 virus infection. Taken together, these findings suggest that *Salmonella* based vaccines represent a promising vaccination approach against influenza viruses and is thus worthy of further investigation.

## Materials and Methods

### Bacterial Strains, Virus, and Cell Line

The bacterial strains used in this study are listed in Supplementary Table S1. Influenza virus A/Puerto Rico/8/34 (H1N1) was used in this study. The virus was cultivated in the allantoic cavity of SPF embryonated eggs, titered in Madin Darby Canine Kidney (MDCK) cells, and expressed as 50% tissue culture infective dose (TCID_50_). The 50% egg infective dose (EID_50_) of H1N1 was determined in SPF embryonated eggs by Reed and Muench method (Reed and Muench, 1938), before use in challenge experiment.

### Construction of an Attenuated Auxotrophic *Salmonella* Typhimurium Mutant Expressing HA1 and M2e Proteins

The optimized HA1 gene sequence of Influenza virus A/Puerto Rico/8/1934 (H1N1) was synthesized (Bionee, Korea) and built into the pJHL65 plasmid, *an asd+* constitutive expression vector, and propagated in *asd* mutated *Escherichia coli* strain as previously described (Hur and Lee, 2011). The recombinant plasmid, pJHL65-HA1, was subsequently transformed into an attenuated auxotrophic mutant of *S*. Typhimurium, strain JOL1800, and the resultant clone was designated as JOL1917 (Sal-HA). The JOL1800 strain was constructed by the deletion of the *lon*, *cpxR*, *asd*, and *wbaP* genes from the wild-type *S.* Typhimurium, JOL401 isolate, as described earlier (Hur and Lee, 2011), and used as the delivery vehicle for the HA1 protein. The synthesized H9N2 M2e gene with sequence (MSLLTEVETPTRNGWECKCSDSSD) was amplified and inserted into the pJHL65 expression vector. Both HA1 and M2e genes were cloned in frame downstream to the β-lactamase signal sequence (*bla* SS) present in the pJHL65 vector to elicit the periplasmic secretion of the expressed proteins in JOL1800 strain. The multiple tandem copies of M2e gene was cloned and expressed as previously described (Kim et al., 2013). Briefly, for the 1 x M2e gene cloning, M2e was amplified using a forward primer containing *EcoR1* site (primer 1) and a reverse primer (primer 3) bearing the *BamH1* and *HindIII* sites with stop codon in between (Supplementary Table S1). To create the M2e dimer, a forward primer containing the *Bgl II* recognition site (primer 2) was used and paired with primer 3. Amplicons from primer1/3 and primer 2/3 were digested with the corresponding *EcoR1/BamH1* (fragment 1) and *BglII/HindIII* restriction enzymes (fragment2), respectively. The fragments were then ligated together with *EcoR1/HindIII* digested pJHL65 expression vector and propagated in *asd* mutated *E. coli* cells. The recombinant plasmid pJHL65-2M2e was further digested with *BamH1*/HindIII and then fused with the fragment 2 to create pJHL-3M2e. The process was repeated until a construct bearing four copies of the M2e gene was produced. The recombinant pJHL65-4M2e plasmid was transformed into JOL1800, and the resultant clone was designated as JOL1913 (Sal-M2e).

To produce the coating antigen for determination of the HA and the M2e specific antibody responses, the HA1 and M2e were cloned into pET28a (+) and pET32a (+) expression vectors, respectively, (Novagen, San Diego, CA, USA), and subsequently transformed into *E. coli* BL21 plys strain (Novagen, USA) for protein expression (Hajam et al., 2013). The expressed proteins both in *S.* Typhimurium and in *E. coli* were confirmed by Western blot analysis using either polyclonal HA antibody (#A01557; GenScript, USA) or polyHis-Tag antibody (#AB-TA13002, AprilBio, Co., Ltd, Korea). The *E. coli* expressed proteins were purified by Ni-NTA chromatographic column and dialysed against PBS (three washes). Purified proteins were quantified by a Bradford assay (Bradford, 1976), filtered, and stored at -20°C until further use.

### Mice Immunization

All animal experimentation work was approved by the Chonbuk National University Animal Ethics Committee (CBNU2015-00085), and was carried out according to the guidelines of the Korean Council on Animal Care and Korean Animal Protection Law, 2007; Article 13 (Experiments with Animals). Four weeks old BALB/c mice were purchased and maintained under standard conditions, and provided antibiotic-free food and water *ad libitum*. One week later, the mice were divided randomly into five groups (*n* = 17). Groups 1 (im-Sal-HA-M2e), 2 (ip-Sal-HA-M2e) and 3 (po-Sal-HA-M2e) were immunized intramuscularly (i.m), intraperitoneally (i.p) and orally (po), respectively, with 2 × 10^7^ colony forming units (CFU) of each JOL1913 and 1917 in 100 μl volume. Groups 4 (Sal-vector) and 5 (PBS) received intramuscularly 100 μl of JOL1837 containing an empty pJHL65 vector and 100 μl of PBS, respectively. The strain JOL1837 is an O antigen deficient JOL1800 strain carrying an empty pJHL65 vector only. Serum samples were collected on the day of immunization (pre-immunization) and weekly thereafter to assess the HA and the M2e specific immune responses. Further, animals (*n* = 4) were sacrificed on days 4 and 14 post-immunization to assess the bacterial load in spleen as described previously (Yin et al., 2015). The animals were observed throughout the period of experiment for any toxicity issues. To evaluate the safety of the vaccine, additional group (*n* = 8) was inoculated intramuscularly with the wild type *S.* Typhimurium, strain JOL990, and safety of the vaccine was recorded in the context of the death of the animals.

### Antigen Specific ELISA

Sera samples were drawn from mice at indicated time points after immunization and before challenge with the influenza virus strain A Puerto Rico/8/34 (H1N1). An indirect ELISA was used to measure the systemic HA and M2e specific IgG1 and IgG2a antibodies in the sera as described previously (Won and Lee, 2016).

### Hemagglutination Inhibition (HI) Assay

Hemagglutination inhibition (HI) assay was performed to assess the HI titers in the sera of immunized and control mice as described previously (Huleatt et al., 2008).

### DC Vaccination

Bone marrow-derived primary dendritic cells (BMDCs) were prepared from BALB/c mice using murine rGM-CSF (Ebensen et al., 2004). For stimulation experiments, approximately 2 × 10^6^/ml BMDCs were cultured in 6 well plates in complete RPMI-1640 media and treated with either heat killed JOL1917 (400 particles/cell) or left unstimulated for 16 h. Then DCs were concentrated by centrifugation, washed, resuspended in sterile PBS, and injected by IP route into female BALB/c mice (2 × 10^6^ per mice, *n* = 3). Second DC vaccination was given after 7 days post-first immunization. Serum samples were collected on day 14 and 21 post-first immunization for determination of HI titers, and isotype specific IgG1 and IgG2a antibodies.

### IFN-γ ELISPOT Assay

The relative numbers of IFN-γ expressing T cells in single cell spleen suspensions were measured using the mouse IFN-γ ELISPOT kit (#88-7314-88, MAB TECH, Sweden) as per the manufacturer’s instructions. Briefly, splenocytes (2 × 10^5^, *n* = 4) were added to each well of pre-coated 96 well plate in triplicate, and stimulated with or without 100 μl/well (10 μg/ml) of a purified HA protein at 37°C for 40 h. After incubation, the cells were removed and the plate was washed and incubated with 100 μl/well (1 μg/ml) of a biotinylated detection antibody (#R4-6A2-biotin) for 2 h at room temperature. Then the plate was washed and incubated with 100 μl/well of Streptavidin-HRP (1:1000) for 1 h at room temperature. Finally, the plate was treated with 100 μl/well of the ready to use TMB substrate solution and incubated until distinct spots emerged, and the reaction was stopped by washing extensively in deionized water. The number of spots was counted in a dissection microscope and the results were expressed as spot forming cells (SFC) per million cells.

### RT-PCR Assay

Splenocytes stimulated with recombinant HA protein were harvested after 24 h, and the total RNA was isolated by RNeasy Mini kit (Qiagen, Hilden, Germany) as per the manufacturer’s instructions. The cDNA was prepared from equal quantity of RNA (1 μg) using SuperScript^TM^ III Reverse Transcriptase kit (Invitrogen, San Diego, CA, USA) as previously described (Hajam et al., 2015), and stored at -20°C until use. Real time PCR assay (qRT-PCR) for gene expression studies was performed with the ABI applied biosystems using Power SYBR Green PCR Master Mix (#4367659, Applied Biosystems, USA) as described previously (Won and Lee, 2016). The relative amounts of cytokine mRNA present (normalized with GAPDH) was determined by 2^-ΔΔCT^ method (Pfaffl, 2001).

### Studies of Immunized Mice Challenged with Lethal H1N1 Virus

For viral challenge experiments, mice (*n* = 9, each group) were first anesthetized with sevoflurane and then challenged intranasally with 50 μl PBS (25 μl per nostril) containing a dose of 10^6^EID50 of lethal influenza virus strain A Puerto Rico/8/34 (H1N1) 4 weeks after vaccination as previously described (Kamble et al., 2017). The infected mice showed the typical effects of systemic infection caused by influenza virus. The mice were observed daily to monitor body weight for 14 days and humane endpoints were used during the survival experiments. Animals were considered gravely ill and were euthanized by overdose of chloroform if they lost more than 30% of their body weight or exhibited lethargy, ruffled hair coat or hunched posture. We have taken special precautions and followed standard guidelines as per the Guide for the Care and Use of Laboratory Animals to minimize sufferings of animals.

For determination of viral titers, lungs were isolated from mice (*n* = 3) at day 4 after virus inoculation. Lung tissues of equal weight were homogenized in DMEM medium to achieve 10-fold serially diluted suspensions of tissue homogenates and were titrated in 96-well culture plates of MDCK cells. The titers were calculated by use of the Reed-Muench method and were expressed as log10 TCID_50_/g lung tissue.

### Statistical Analysis

Statistical analysis was performed using GraphPad prism 7.00 program (San Diego, CA, USA). Data were analyzed by two tailed unpaired student’s *t*-test to compare the data from gene expression studies. One way ANOVA with Tukey’s multiple comparison test was used between different groups. Data are represented as mean ± standard deviation. *p* < 0.05 were considered statistically significant.

## Results

### Design of an Influenza HA and M2e Based Vaccine Delivered by an O Antigen Deficient *Salmonella* Mutant

**FIGURE 1:**
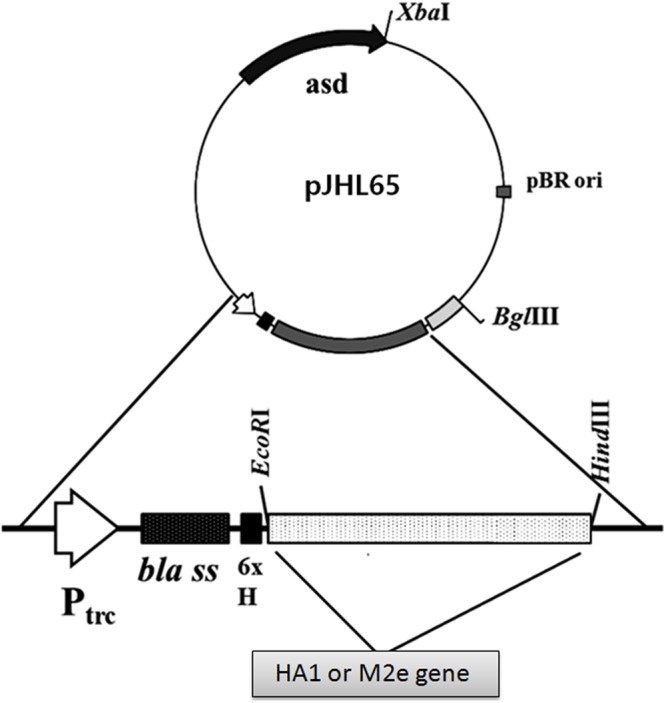
**Construction of a pJHL65 recombinant plasmid expressing either HA1 or M2e gene.** The codon optimized HA1 or M2e gene was cloned in frame downstream to the *bla* signal sequence of pJHL65 vector at *EcoR1* and *HindIII* site, and the presence of respective genes in recombinant pJHL65 plasmid were confirmed by colony PCR and RE analysis.

The synthetic HA1 of Influenza virus A/Puerto Rico/8/1934 (H1N1) and M2e genes were codon optimized for expression in *S*. Typhimurium. The HA1 and M2e genes were amplified using gene specific primers and cloned into pJHL65, an *asd*+ constitutive expression vector, respectively, and the clones were propagated in *Δasd E. coli* strain as described previously (Hur and Lee, 2011). To direct the expressed proteins to the periplasmic space, the genes were cloned in frame downstream to the *bla* SS of the pJHL65 vector (**Figure 1**). The insertion of HA1 or M2e gene into pJHL65 vector was confirmed by digestion of the positive clones with *EcoR1* and *HindIII* to release a fragment of 675 bp or 396 bp sizes. Subsequently, the pJHL65-HA1 gene construct or pJHL65-M2e plasmid was electroporated into a *ΔcpxR*, *Δlon*, *Δasd*, and *ΔwbaP* mutated *S*. Typhimurium strain, JOL1800, and the resultant clones were designated as JOL1917 (Sal-HA) and JOL1913 (Sal-M2e), respectively. The strain JOL1800 is an O antigen deficient mutant derived from the strain JOL912 as previously described (Hur and Lee, 2011). The O antigen of JOL912, encoded by *wbaP*, was deleted by the allelic exchange method (Hur and Lee, 2011) and the silver-staining analysis confirmed the deletion of the O antigen. The silver staining verified the typical LPS ladder of the polymeric O antigen for the wild type, and the absence of this pattern in the JOL1800 strain (Data not shown). Western blot analysis showed a protein band corresponding to the 28 kDa and the 16 kDa, the expected size of our proteins and thus confirmed the expression of HA and M2e proteins in JOL1800, respectively (**Supplementary Figure S1**). The proteins were biologically active as evidenced by the elicitation of the antigen-specific immune responses in mice, as observed in the present study.

### Bacterial Recovery from Immunized Mice and *In Vivo* Toxicity Assay

**FIGURE 2:**
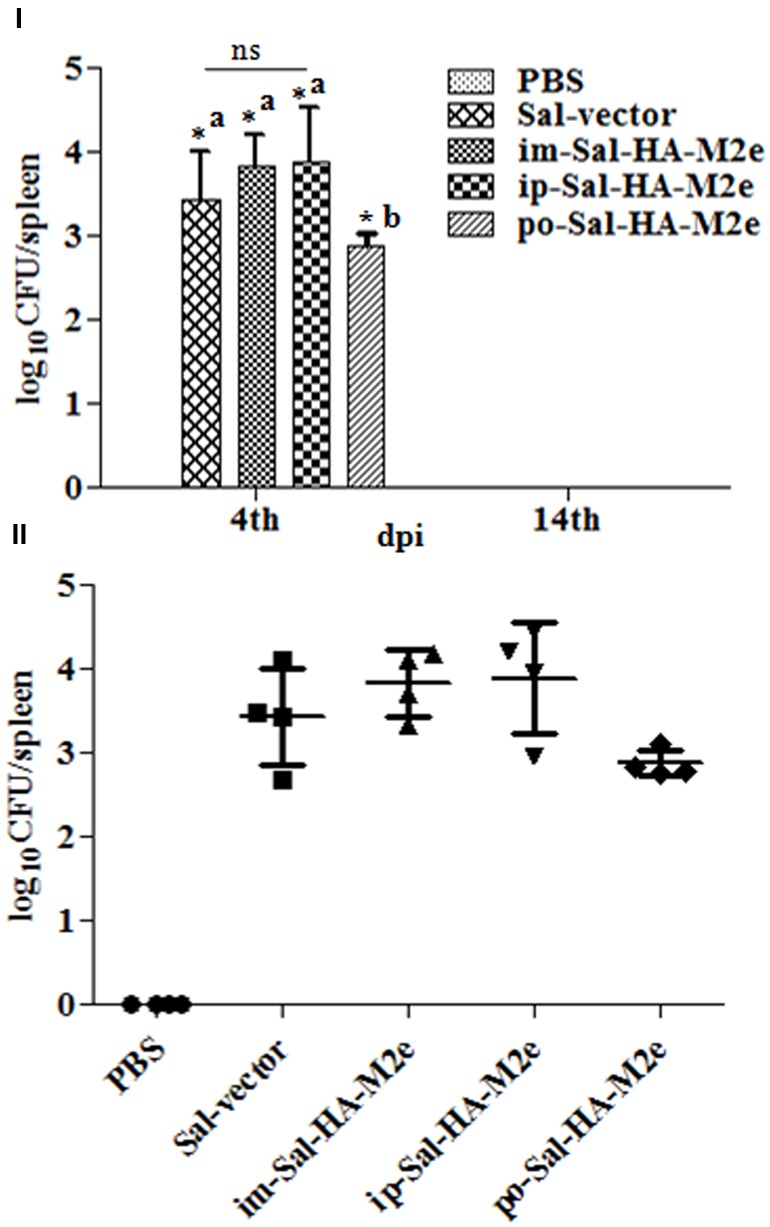
**Bacterial recovery from infected mice.** Mice were either inoculated intramuscularly with the PBS only, JOL1837 carrying empty vector (Sal-vector) and constructs JOL1917+1913 (im-Sal-HA-M2e) or intraperitoneally with constructs JOL1913+1917 (ip-Sal-HA-M2e) or orally with constructs JOL1913+1917 (po-Sal-HA-M2e). After 4 and 14 days post-immunization (dpi), 4 animals in each group at each time point were sacrificed and the bacterial load in spleen was evaluated. **(I)** Mean bacterial count of different groups and the results are expressed as log_10_ CFU/spleen. Each data point represents mean of four animals. **(II)** Bacterial counts of individual animals of each group at 4th dpi and the results are expressed as log_10_ CFU/spleen. ^∗^*p* < 0.05; ^a^significant with respect to PBS, Sal-vector and po-Sal-HA-M2e groups; ^b^significant with respect to Sal-vector and PBS controls; dpi, days post-immunization; ns, non-significant.

**FIGURE 3:**
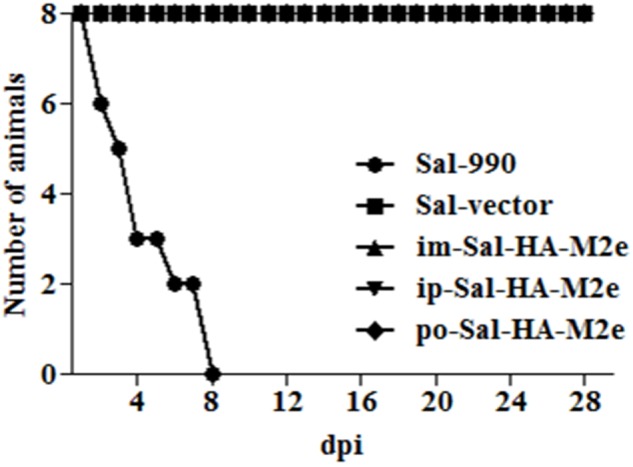
***In vivo* safety evaluation of the vaccine.** Mice were either inoculated intramuscularly with the wild type strain JOL990 (Sal-990, *n* = 8), PBS only, JOL1837 carrying empty vector (Sal-vector) and constructs JOL1917+1913 (im-Sal-HA-M2e) or intraperitoneally with constructs JOL1913+1917 (ip-Sal-HA-M2e) or orally with constructs JOL1913+1917 (po-Sal-HA-M2e). The animals were monitored for 28 days and the mortality of the animals was recorded. Non-lethal endpoints were used during the experiments and mice were euthanized to limit suffering, when they exhibited weight loss of more than 30%, lethargy, ruffled hair coat, or hunched posture.

To evaluate the bacterial load in mice immunized with either JOL1913+1917 (2 × 10^7^cfu each/mouse), JOL1837 (4 × 10^7^ cfu/mouse) or PBS (100 μl), four mice from each group were sacrificed at 4 and 14th day post-immunization (dpi), and bacterial count in spleen was estimated. Mice immunized with attenuated *S*. Typhimurium showed mean bacterial counts of 2.87–3.89 logs CFU/spleen at 4th dpi, independently of the route of vaccination, while no bacterial count was recorded at 14th dpi (**Figure 2I**). This finding demonstrates that attenuated *Salmonella* delivering heterologous antigens efficiently reach to the secondary lymphoid organs. This increases the likelihood of antigen-specific lymphocytes encountering their cognate antigens and subsequent elicitation of immune responses against the *Salmonella* derived foreign antigens. Among *Salmonella* vaccinated groups, orally immunized mice group showed significantly (*p* < 0.05) lower bacterial counts in spleen compared to the parenterally immunized groups, which showed almost comparable bacterial counts (**Figures 2I,II**). Further, mice were closely monitored for any signs of toxicity. All mice infected with JOL1913+I917 and JOL1837 remained alive throughout the period of experiment. In contrast, mice inoculated with a much lower dose of the wild type strain, JOL990 (1 x10^6^), died within a period of 7 days (**Figure 3**). Thus, the constructed JOL1917+JOL1913-based vaccine appeared to be relatively safe *in vivo* as compared to the wild type strain.

### Sal-HA-M2e Based Vaccine Stimulates Efficient Antibody Responses

**FIGURE 4:**
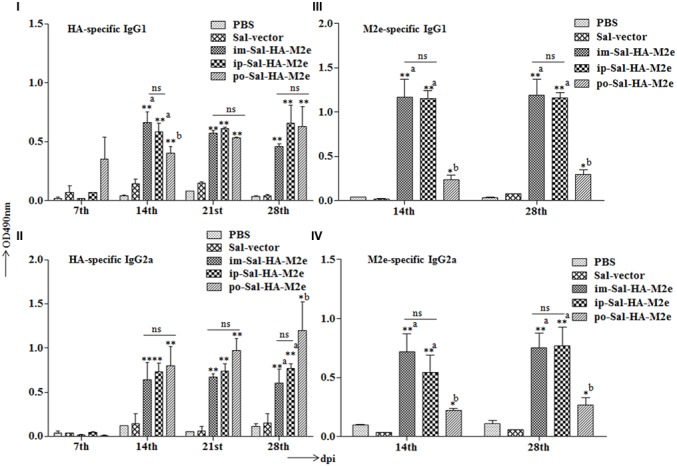
**Efficient humoral immune responses are stimulated in BALB/c mice (*n* = 6) after vaccination with JOL1913+1917.** Mice were either immunized intramuscularly with the PBS only, JOL1837 carrying the empty vector pJHL65 (Sal-vector) and constructs JOL1913+JOL1917 (im-Sal-HA-M2e), or intraperitoneally with JOL1913+JOL1917 (ip-Sal-HA-M2e) or orally with constructs JOL1913+1917 (po-Sal-HA-M2e). The antibodies IgG1 and IgG2a were measured in serum at different time points post-vaccination by indirect ELISA. **(I,II)** Kinetics of HA-specific IgG1 and IgG2a responses, respectively, in sera from vaccinated mice. **(III,IV)** Kinetics of M2e-specific IgG1 and IgG2a responses, respectively, in sera from vaccinated mice. The assays are performed in duplicate and the data are presented as mean ± SD. ^∗^*p* < 0.05, ^∗∗^*p* < 0.01; ^a^significant with respect to PBS, Sal-vector and po-Sal-HA-M2e groups; ^b^significant with respect to Sal-vector and PBS controls; dpi, days post-immunization; ns, non-significant.

To examine the ability of Sal-HA-M2e-based vaccine to induce humoral responses in BALB/c mice, isotype-specific antigen ELISA was carried out to assay the levels of serum IgG1 and IgG2a at 0,7,14, 21, and 28th dpi. The kinetics of HA and M2e specific IgG1 and IgG2a responses are shown in **Figure 4**. Our results demonstrated that the mice immunized with *Salmonella*-HA-M2e based vaccine displayed significantly higher (*p* < 0.05) HA and M2e-specific IgG1 and IgG2a responses compared to the controls. The induction of IgG1 and IgG2a responses were independently of the route of vaccination. Both the HA and the M2e specific IgG1 and IgG2a levels were detected at 14th dpi that maintained till 28th dpi in all the Sal-HA-M2e immunized groups (**Figures 4I–IV**). Our results demonstrated that Sal-HA-M2e immunized groups showed almost comparable levels of HA-specific IgG1 and IgG2a responses (**Figures 4I,II**). However, the M2e-specific IgG1 and IgG2a responses were significantly (*p* < 0.05) lower in orally vaccinated group as compared to the im-Sal-HA-M2e and ip-Sal-HA-M2e immunized groups (**Figures 4III,IV**). Further, the ratio of IgG1/IgG2a suggested that coadministration of Sal-HA and Sal-M2e in mice skewed the immune response toward Th1 type against HA while as Th2 type against M2e. This demonstrates that the strategy of coadministration of Sal-HA and Sal-M2e strains has potential to elicit efficient HA and M2e specific humoral immune responses in mice.

**FIGURE 5:**
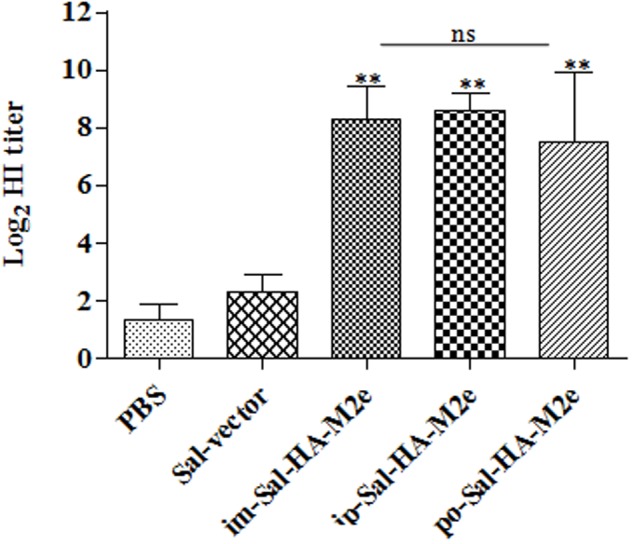
**Specific antibody titers in mice (*n* = 6) after vaccination with JOL1913+1917.** Mice were either immunized intramuscularly with the PBS only, JOL1837 carrying the empty vector pJHL65 (Sal-vector) and constructs JOL1913+JOL1917 (im-Sal-HA-M2e), or intraperitoneally with JOL1913+JOL1917 (ip-Sal-HA-M2e) or orally with constructs JOL1913+1917 (po-Sal-HA-M2e). Serum samples were analyzed for HI activity after 28 days post-vaccination. Each data points represent mean ± S.D. ^∗∗^*p* < 0.01; ns, non-significant.

The functional activities of the sera from the vaccinated mice were further investigated by determining the HI titers against the influenza virus strain A Puerto Rico/8/34 (H1N1). Consistent with our results of the serum IgG1 and IgG2a responses, mice immunized with Sal-HA-M2e based vaccine also displayed significantly higher (*p* < 0.01) HI titers as compared to the control Sal-vector and PBS groups (**Figure 5**), independently of the route of immunization. The HI titers were almost comparable in all the Sal-HA-M2e immunized groups. All these findings clearly demonstrate that *Salmonella* based influenza vaccines have potential to induce functional antibodies that confer protective immunity against the influenza infections (Chen et al., 2008).

### *Salmonella*-HA-M2e Based Vaccine Stimulates Efficient CMI Responses

**FIGURE 6:**
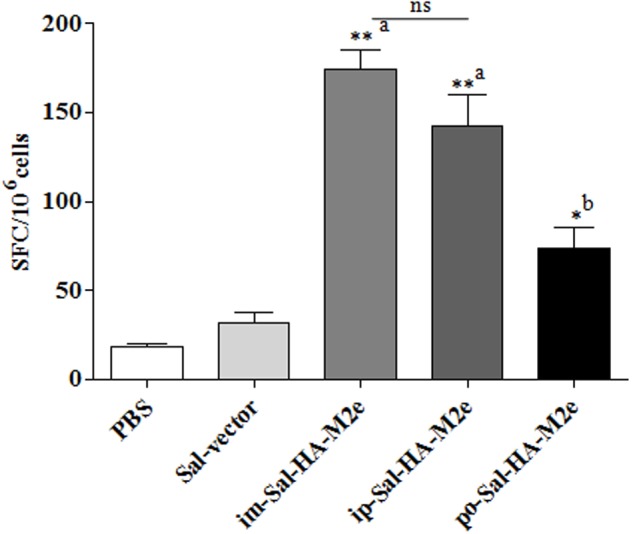
**Analysis of IFN-γ production by HA-specific T cells in immunized mice (*n* = 4) at 14th day after primary immunization.** Mice were either inoculated intramuscularly with the PBS only, JOL1837 carrying empty vector (Sal-vector) and constructs JOL1917+1913 (im-Sal-HA-M2e) or intraperitoneally with constructs JOL1913+1917 (ip-Sal-HA-M2e) or orally with constructs JOL1913+1917 (po-Sal-HA-M2e). Splenocytes (2 × 10^5^) were harvested from immunized mice and restimulated with HA antigen (10 μg/ml) for 40 h. The HA-specific T cells secreting IFN-γ were analyzed by ELSIPOT assay Kit. The results are expressed as spot-forming cells (SFC) per million cells and the assays were performed in duplicate. Each data points represent mean ± SD. ^∗^*p* < 0.05; ^∗∗^*P* < 0.01; ^a^significant with respect to PBS, Sal-vector and po-Sal-HA-M2e groups; ^b^significant with respect to Sal-vector and PBS controls; ns, non-significant.

To study the HA-specific T cell responses induced by the attenuated *Salmonella* based vaccine, splenocytes were isolated from vaccinated mice 14 days after immunization and stimulated with a purified HA protein (10 μg/ml) for 40 h. Our results showed that Sal-HA-M2e vaccination induced significantly higher (*p* < 0.05) anti-HA IFN-γ secreting T cell responses compared to the PBS and the Sal-vector groups (**Figure 6**). However, among Sal-HA-M2e vaccinated groups, orally immunized mice showed significantly (*p* < 0.05) lower anti-HA IFN-γ secreting T cell responses compared to the intramuscularly and the intraperitoneally vaccinated mice groups. These results thus clearly indicate that *Salmonella*-based vaccines induce antigen-specific T cell responses.

**FIGURE 7:**
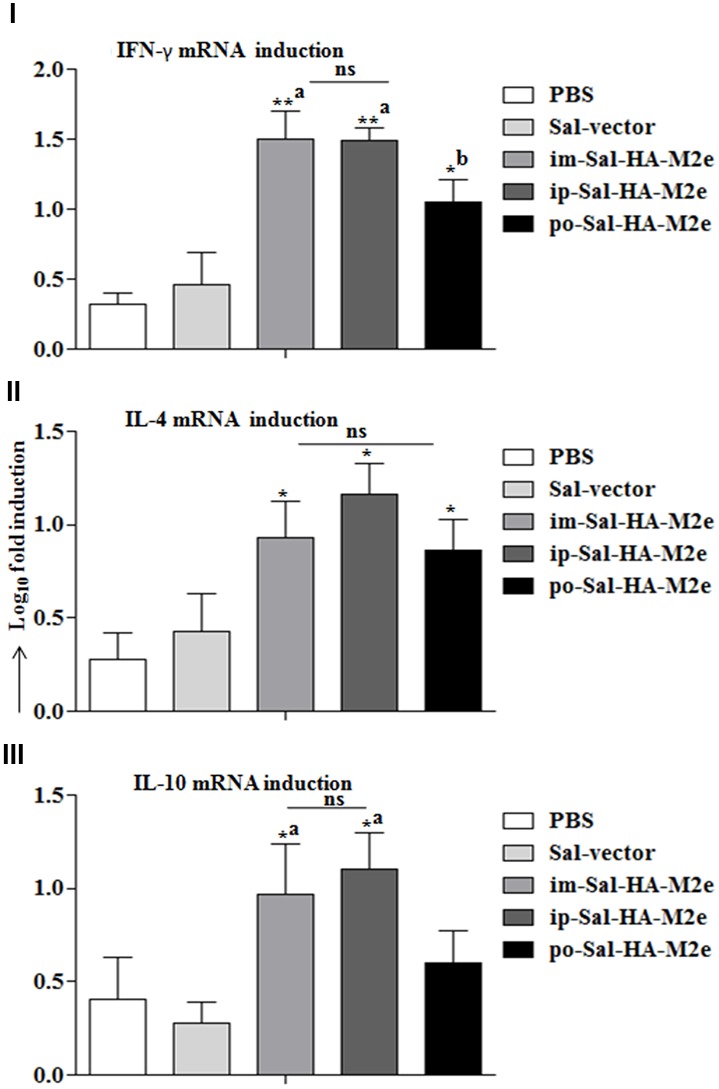
**Analysis of cytokine gene expressions in splenocytes.** Mice were either inoculated intramuscularly with the PBS only, JOL1837 carrying empty vector (Sal-vector) and constructs JOL1917+1913 (im-Sal-HA-M2e) or intraperitoneally with constructs JOL1913+1917 (ip-Sal-HA-M2e) or orally with constructs JOL1913+1917 (po-Sal-HA-M2e). Splenocytes (2 × 10^6^, *n* = 4) were harvested from immunized mice after 14 days post-immunization and restimulated with HA antigen *in vitro* (10 μg/ml) for 24 h. Then the total RNA was extracted and gene transcription for IFN-γ **(I)**, IL-4 **(II)**, and IL-10 **(III)** cytokines was quantified by qRT-PCR assay. Results are expressed as fold induction (log_10_) of cytokine mRNA transcription by HA stimulated cells compared to the media treated (Med) cells. B-actin was used as an internal control and mRNA levels at 0 h was used as calibrator. Histograms represent mean cytokine levels and bars represent SD. ^∗^*p* < 0.05; ^∗∗^*p* < 0.01; ^a^significant with respect to PBS, Sal-vector and po-Sal-HA-M2e groups; ^b^significant with respect to Sal-vector and PBS controls; ns, non-significant.

Next we evaluated the ability of immunized splenocytes to respond to a recall HA antigen in the context of inductions of cytokine gene expressions. In this regard, splenocytes isolated from vaccinated mice 14 days after immunization were stimulated with HA protein (10 μg/ml) for 24 h and then RNA was isolated for quantification of IL-4, IL-10, and IFN-γ mRNA level inductions by qRT-PCR assay. After stimulation with HA protein, splenocytes of Sal-HA-M2e immunized groups showed significantly higher (*p* < 0.05) mRNA induction levels of IFN-γ in comparison to the Sal-vector and the PBS control groups (**Figure 7**). However, mice orally immunized with Sal-HA-M2e vaccine showed significantly (*p* < 0.05) lower IFN-γ mRNA induction levels compared to the im-Sal-HA-M2e and ip-Sal-HA-M2e immunized groups. This result was consistent with the findings of the anti-HA IFN-γ secreting T cell responses observed in the immunized mice. The mRNA levels of IL-4 and IL-10, the Th2 cytokines, were also significantly higher (*p* < 0.05) in Sal-HA-M2e immunized groups compared to the Sal-vector and the PBS control groups (**Figure 7**). The levels of IL-4 were almost comparable in all the Sal-HA-M2e immunized groups while the levels of IL-10 were significantly (*p* < 0.05) higher in i.m and i.p Sal-HA-M2e groups compared to the orally vaccinated group. Further, the IFN-γ mRNA levels were significantly higher (*p* < 0.05) compared to IL-4 and IL-10 mRNA levels, suggesting that *Salmonella* based vaccines have potential to skew the immune response toward Th1 type, which is important for the clearance of influenza infections (McMichael et al., 1983).

### Immunization with DCs Stimulated *Ex Vivo* with JOL1917 Elicits HA-Specific Immune Responses

**FIGURE 8:**
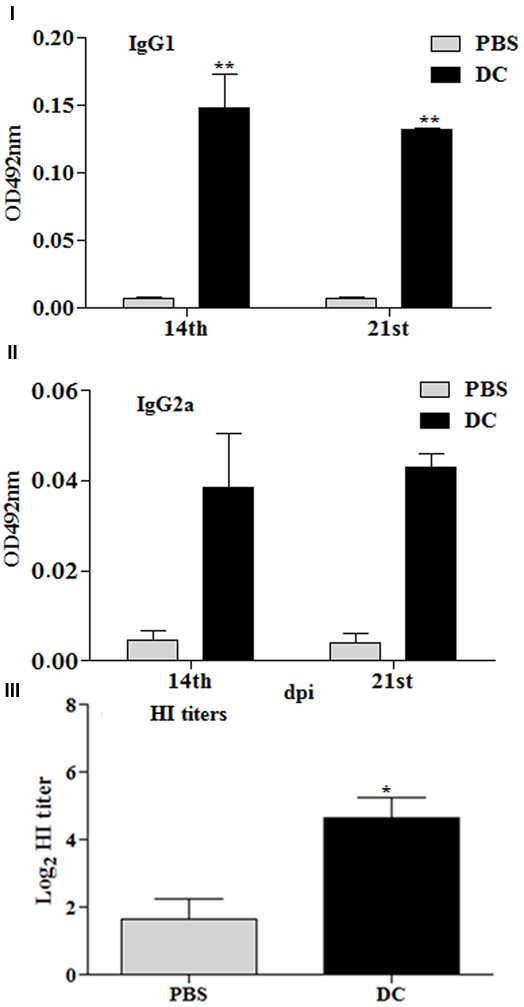
**Immune responses stimulated by DCs stimulated *ex vivo* with heat killed JOL1917.** Murine BMDCs (2 × 10^6^) were stimulated with either JOL1917 (400 particles/cell) or left unstimulated for 16 h. These *ex vivo* stimulated DCs were then injected intraperitoneally in BALB/c mice (*n* = 3) to evaluate the HA-specific immune responses. **(I)** HA-specific serum IgG1 responses 14 and 21 days post-first immunization. **(II)** HA-specific serum IgG2a responses 14 and 21 days post-first immunization. **(III)** HI titers after 14th day post-first immunization. Each data point represents mean ± standard deviation (SD).^∗^*p* < 0.01; ^∗∗^*p* < 0.01.

Additional studies were conducted to gain insights on the role played by DCs in the immune responses stimulated using *Salmonella* as an antigen delivery system. Mice were immunized by IP route with 2 × 10^6^ BMDCs, which had been stimulated *ex vivo* with inactivated JOL1917 (400 particles/cell) for 16 h. Anti-HA antibody responses were detected in all the vaccinated mice (**Figures 8I–III**). *Ex vivo* stimulated DCs induced both IgG1 and IgG2a antibodies in serum, but the shift toward a more dominant Th2 (IgG1) response pattern was obtained using inactivated JOL1917 as a delivery system. In contrast, mice immunized with live attenuated JOL1917 strain showed a shift toward a more dominant Th1 response, as evidenced by the higher inductions of IgG2a levels. This suggests that it is possible to modulate the major Th immune response using *Salmonella* as an antigen delivery system. Further, we evaluated the HI titers in the immunized sera. Our data showed that *ex vivo* stimulated DCs had elicited HI titres in immunized mice as compared to the unimmunized control group (**Figure 8III**). These observations clearly indicate that DCs play an important role in the elicitation of immune responses when attenuated *Salmonella* mutants are used as a technology platform for the delivery of heterologous antigens.

### *Salmonella*-HA-M2e Based Vaccination Provided Significant Protection against Lethal H1N1 Challenge

**FIGURE 9:**
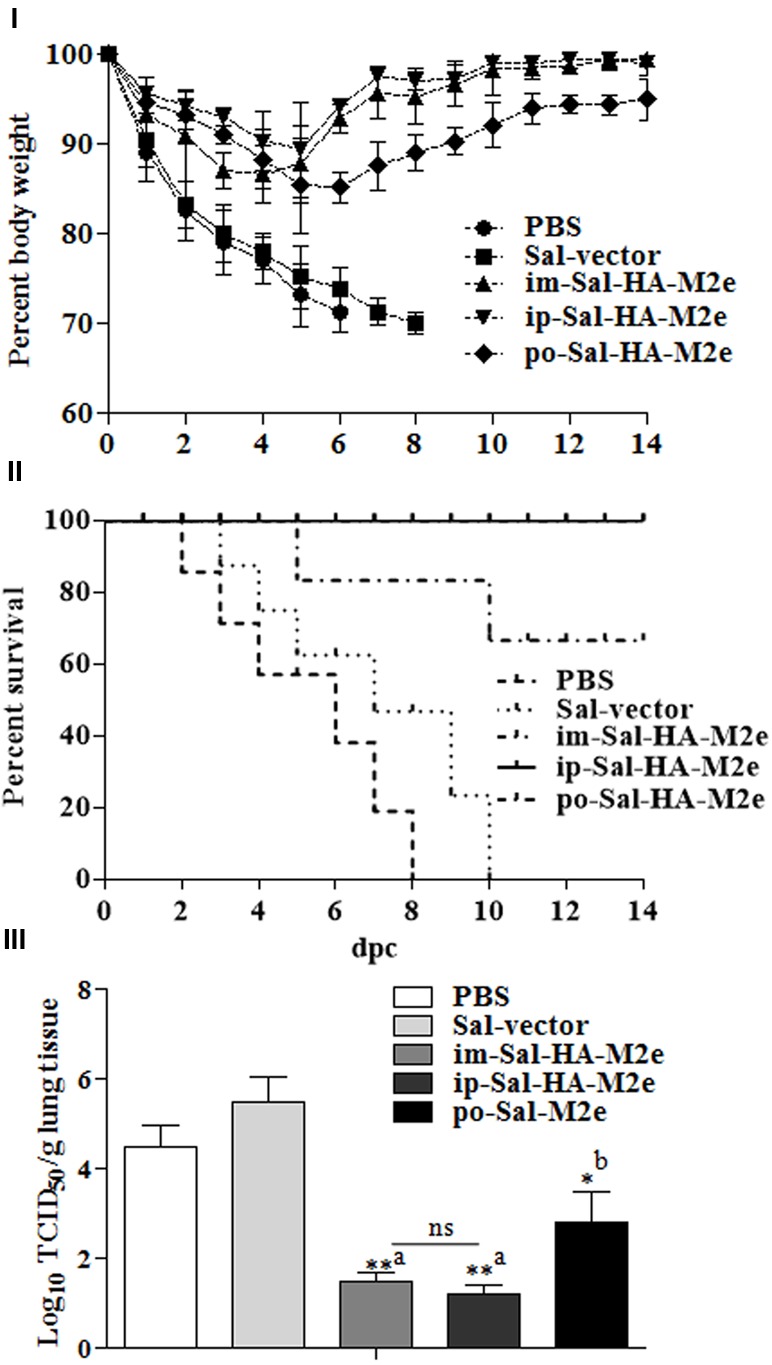
**Immune protection of mice against lethal influenza A/Puerto Rico/8/34 (H1N1) challenge (*n* = 10).** Mice were either inoculated intramuscularly with the PBS only, JOL1837 carrying empty vector (Sal-vector) and constructs JOL1917+1913 (im-Sal-HA-M2e) or intraperitoneally with constructs JOL1913+1917 (ip-Sal-HA-M2e) or orally with constructs JOL1913+1917 (po-Sal-HA-M2e). Mice were then challenged intranasally with 10^6^EID50 of lethal H1N1 virus at 4 weeks after primary immunization. Mice were monitored for weight loss **(I)** and survival **(II)** throughout a 14 day observation period. The results are presented in terms of percent of body weight and percent survival, respectively. **(III)** Lung tissues (*n* = 3) were harvested at 4 days post-challenge (dpc) and viral titers were determined by Reed and Muench method. The experiments were performed in triplicate and the results are presented as mean ± SD. ^∗^*p* < 0.05; ^∗∗^*p* < 0.01; dpc, days post-challenge; ^a^significant with respect to PBS, Sal-vector and po-Sal-HA-M2e groups; ^b^significant with respect to Sal-vector and PBS controls; ns, non-significant.

The benchmark of an influenza vaccine is protection against a lethal virus challenge. Therefore to evaluate the efficacy of Sal-HA-M2e vaccination, all vaccinated and control mice were challenged with a lethal dose of highly pathogenic H1N1 virus at 28th dpi. The clinical protection observed in the context of body weight and survival is shown in **Figures 9I,II**. Immediately after virus challenge, all mice experienced a decrease in body weight. The mice vaccinated with Sal-HA-M2e, independently of the route of vaccination, gradually recovered after day 6 and showed protection in 66.66% orally, 100% intramuscularly, and 100% intraperitoneally immunized mice against a homologous H1N1 virus. The mice vaccinated with either JOL1800 or PBS only, however, showed continued significant weight loss and exhibited no protection against H1N1 challenge (**Figures 9I,II**). The obtained results clearly suggest that Sal-HA-M2e based vaccine provides significant immune protection against the lethal H1N1 influenza virus.

Further, we investigated the ability of Sal-HA+Sal-M2e vaccination on the effect of local replication of challenged virus in immunized and control mice. To this end, lung tissues were harvested from animals at 4th day post-challenge (dpc) and the virus titers in these tissues were determined. The titers of H1N1 virus in mice immunized with JOL1913+1917 were significantly (*p* < 0.05) lower than those in animals administered with either PBS or Sal-vector (**Figure 9III**). The titers were 1000-fold lower in case of i.m-Sal-HA-M2e and i.p-Sal-HA-M2e immunized groups, and less than 1000-folds in case of po-Sal-HA-M2e immunized group. These findings are consistent with our immune protection results, and thus clearly indicate that *Salmonella*-based vaccines can induce immune protection against the highly pathogenic influenza A virus challenges.

## Discussion

Our goal was to investigate whether a novel attenuated O antigen deficient *Salmonella* mutant delivering HA and M2e could elicit a significant HA and M2e specific immune responses, and subsequent protection in mice against a lethal challenge of H1N1 influenza A virus. A major obstacle in vaccine development against influenza viruses is the extent of genetic diversity, and consequently, antibodies raised against one vaccine strain provide little or no protection against the heterologous strain (Nicholson et al., 2003; Deng et al., 2015). One approach to induce a broad spectrum of protection is to design a vaccine that is based on the highly conserved region of a virus subjected to minor antigenic variations. M2e is an attractive choice to elicit a broad spectrum of protection against many subtypes as it is highly conserved target for the universal development of influenza A vaccine (Wang B.-Z. et al., 2012; Deng et al., 2015; Berlanda Scorza et al., 2016). However, when presented by itself, is weakly immunogenic and remarkably, natural infection induced anti-M2e responses are very modest, most likely due to its low presence on virions (Deng et al., 2015). Several strategies have been employed to enhance the M2e-specific antibody responses, for instance, linking of M2e to a suitable carrier (Layton et al., 2009) or increasing the density of M2e molecules by expressing multiple tandem copies of M2e (Deng et al., 2015). Although M2e-specific antibodies were enhanced and certain level of cross protection was observed in animal models, but complete protection against the lethal infections was not achieved (Layton et al., 2009; Deng et al., 2015). Previously, *Salmonella* system has been used to deliver M2e in chickens and significant protection against low pathogenic avian influenza (AI) H7N2, but not highly pathogenic H5N1 AI was observed (Layton et al., 2009). Combining HA and M2e is an attractive approach to induce broadly reactive anti-M2e specific antibodies. Previous study shows that immunization with M2e peptide formulated in Freund’s adjuvant failed to elicit M2e-specific antibody responses, but by including a T cell epitope derived from HA in the M2e conjugate, M2e-specific antibodies were readily induced (Pejoski et al., 2010). Further, the degree of M2e epitope density linked to the carrier molecule is a critical factor for the elicitation of efficient and protective M2e-specific immune responses (Zebedee and Lamb, 1988; Grandea et al., 2010). In accordance with this notion, we coadministered attenuated *Salmonella* strains expressing HA and multiple tandem copies of M2e, respectively, in mice and evaluated the potential of this strategy to elicit the HA and the M2e specific immune responses. Induction of HA-specific neutralizing antibody responses in peripheral blood circulation strongly correlates with the recovery from the clinical disease and protection from subsequent challenge infections (Chen et al., 2008). The present study demonstrates that the Sal-HA*-*M2e based vaccine induced significantly elevated levels of HA-specific antibody responses and subsequent protection against H1N1 challenge. The induction of antibody responses was independent of the route of vaccination, and consistently the protection against infection was also independent of the route of immunization. The type of HA and M2e specific IgG isotypes that are elicited by immunization correlates well with the protective effectiveness of the vaccine in mice (Viertlboeck et al., 2007; Pejoski et al., 2010). Although T cell responses induced by an M2e-based vaccine have been reported previously, the protection by the M2e vaccination is mainly mediated by the induced antibodies but not the T cells (Deng et al., 2015). In the present study, we show that *Salmonella* delivering HA and M2e proteins induced significantly higher HA and M2e specific IgG1 and IgG2a responses and this correlates well with the immune protection studies. We found complete protection against H1N1 challenges in intramuscularly and in intraperitoneally immunized groups, which showed significantly higher both HA and M2e-specific humoral immune responses. However, the M2e-specific humoral responses were significantly lower in orally vaccinated mice group compared to the im-Sal-HA-M2e and the ip-Sal-HA-M2e immunized groups. This result might explain why immune protection was comparatively less in case of orally immunized mice group. Our results are in accord to the previously published report that shows mice orally immunized with a *S.* Typhimurium delivering influenza nucleoprotein resulted in comparatively less immune protection than the intranasally and the intraperitoneally immunized mice groups (Ashraf et al., 2011). Previous study has also reported enhanced immune responses induced by the multiple repeats of the M2e and the HA delivered through the adenoviral vectors (Kim et al., 2014). The potential of this approach has been demonstrated by inducing HA-specific and broadly cross reactive M2-specific antibodies and significant protection against lethal live virus challenge as shown in the present study.

The cell mediated immunity induced by influenza viruses are necessary for viral clearance from the lungs and are, therefore, critical for mice to recover from influenza virus infections. In the present study, the Sal-HA-M2e based vaccine not only generated efficient humoral immune responses but also induced significantly higher anti-HA specific IFN-γ T cell responses. This finding is consistent with the previous report that a DNA vaccine encoding M2e together with H1 HA induced significantly higher HA-specific CD8+ and M2e-specific T cell responses compared to the DNA vaccine encoding M2e or H1 HA alone (Wang B. et al., 2012). This can be explained, in part at least, by *Salmonella* dependent improvement in the capacity of DCs to process and present both MHC class-I and II restricted Ags. The immunization studies performed with the *ex-vivo* DCs loaded with heat killed JOL1917 suggest that DCs play an important role in the elicitation of immune responses when *Salmonella* are used as a foreign antigen delivery system. DCs loaded with heat-killed JOL1917 elicited antigen specific immune responses as evidenced by the significantly higher induction of HA-specific IgG1 and IgG2a responses. Further, *ex vivo* stimulated DCs elicited HI titers against the influenza virus, suggesting that DCs play an important role in triggering a specific immune response during *Salmonella* infections. Our results are in accord to the previously published report (Yrlid et al., 2001). These findings clearly indicate that *Salmonella* expressing foreign antigens are biologically active and promote antigen-specific immune responses.

Further, we evaluated the recall cytokine responses in splenocytes. The nature of the cytokines released during the activation process is an important parameter to define the type of immune response elicited. Our data demonstrated that there is an increment in IFN-γ, a Th1 cytokine, expressions by HA restimulated splenocytes. IFN-γ is a prototypic Th1-polarizing cytokine and is very critical in the development of cellular immune responses, especially cytotoxic CD8+ responses, which are effective in clearance of viral infections (Matteoli et al., 2008; Mills, 2008). We also observed an increment in IL-10 and IL-4, the Th2 cytokines, which have a clear role in the generation of potent antibody responses through class switching to IgG1 and IgG3 (Moore et al., 1993; Sanders et al., 2006). The induction of INF-γ was significantly higher than IL-10 and IL-4, suggesting that the present strategy results in a mixed type of immune response with preferred Th1 type shift. Interesting, we have also observed an increment in the number of influenza-specific IFN-γ producing T cells in Sal-HA-M2e immunized mice, in response to restimulation with a recombinant HA protein. IFN-γ is a critical cytokine in the generation of potent cytotoxic CD8+ T cell responses, which have potential to provide non-specific protection against the pathogenic viruses (McMichael et al., 1983; Sridhar et al., 2013). The CD8+ T cell responses are essential to clear influenza viruses from the lungs and, having their broad antigen-specificity, can reduce disease symptoms caused by a potential pandemic influenza A virus outbreak (McMichael et al., 1983; Sridhar et al., 2013). Indeed, there exists a strong correlation between productive virus infection and cross-reactive cellular immunity against subsequent infection with heterologous influenza viruses based on the studies involving humans and other host-species (McMichael et al., 1983; Kreijtz et al., 2007; Sridhar et al., 2013). These findings clearly indicate that *Sal*-HA-M2e based vaccine has the potential to stimulate efficient protective humoral and CMI responses and can protect from pathogenic influenza infections. Our results are in accord to the previously published report that shows *S.* Typhimurium delivering influenza HA and NA proteins elicited efficient IFN-γ responses and significant protection against influenza virus infections (Pei et al., 2015). The present study also demonstrated that orally immunized mice group resulted in lower IFN-γ responses compared to the intramuscularly and the intraperitoneally immunized mice groups, which might explain the immune protection observed in the present study.

*Salmonella* based vaccines have certain advantages over conventional influenza vaccines. These vaccines are readily amenable to modifications once the circulating strains are identified and can be rapidly produced in large quantities without the need of specific cell culture conditions, as is the case with whole virus based influenza vaccines. Moreover, the delivery of foreign antigens by *Salmonella* provides an appropriate danger signals to the immune system, acting as natural adjuvant, thereby promoting efficient maturation and activation of DCs, which is prerequisite for the induction of potent adaptive immune responses (Ebensen et al., 2004; Sharpe, 2009). Previously, attenuated *Salmonella* system has been used to deliver HA based DNA vaccines against highly pathogenic H5N1 influenza virus, and partial protection was conferred against the virus infection (Van et al., 2013). A potential limitation with the DNA vaccines delivered through bacteria is the inability to stably maintain the plasmid DNA inside the bacteria. Moreover, the gene expression of heterologous antigens encoded by DNA is inefficient, resulting in the poor immunogenicity of the vaccine (Van et al., 2013). Our strategy of employing *Salmonella* system expressing HA and M2e proteins has elicited significant immune protection against the lethal H1N1 challenge infection. The removal of the immunodominant O-antigen makes the vaccine strain relatively safe as demonstrated in this study. Furthermore, the outer-membrane proteins and other foreign antigens carried by the O-antigen deficient *Salmonella* mutant are more exposed to the immune system (Kong et al., 2011), which in turn might result in the enhanced immunogenicity against the *Salmonella*-derived membrane proteins.

Taken together, our strategy of employing the codon-optimized HA and M2e amino acid sequences delivered by an auxotrophic mutant of *S*. Typhimurium has proven to be effective in eliciting a significant protective immunity in mice. Our results of the HA and the M2e specific immune responses elicited by Sal-HA-M2e vaccine suggest that attenuated *Salmonella* system simultaneously carrying expression cassettes for various antigens can function as a vaccine candidate against multiple strains of influenza viruses. Thus, *Salmonella* based vaccines constitute a promising technology for the development of more efficient multivalent protein based vaccines. Considering the potential of M2e specific immunity to virus, the present strategy may offer cross protective immunity against different subtypes of influenza A viruses, and thus warrants further investigation in this regard. Further, additional studies are needed to study the effect of *Salmonella*-based influenza vaccines in other animal models that should facilitate the development of *Salmonella*-based vaccines against influenza virus infection.

## Author Contributions

JL and IH conceived and designed the work. IH did the experiments. IH wrote the manuscript and statistical analysis. JL did the critical revision and provided necessary support to carry out the experiments.

## Conflict of Interest Statement

The authors declare that the research was conducted in the absence of any commercial or financial relationships that could be construed as a potential conflict of interest. The reviewer VC and handling Editor declared their shared affiliation, and the handling Editor states that the process nevertheless met the standards of a fair and objective review.

## Supplementary Material

FIGURE S1 **Western blot analysis of HA and M2e proteins expressed by JOL1800 strain.** The HA1 and multiple tandem copies of M2e proteins expressed by JOL1800 strain were confirmed by Western blot analysis. The JOL1800 bacteria harboring either pJHL65-HA1 plasmid (JOL1917), pJHL65-M2e plasmid (JOL1913) or empty pJHL65 plasmid (JOL1837) were allowed to grow till 0.6 OD_600nm_. Then bacterial pellets were subsequently subjected to Western blot analysis using poly His-Tag antibody (#AB-TA13002, AprilBio, Co., Ltd, Korea). Lane M, protein Marker (#P8500, GenDEPOT, USA); lane 1, bacterial pellet of JOL1913 showing a 16 kDa band; lane 2, bacterial pellet of JOL1917 showing a 28 kDa band; lane 3, control JOL1837 bacterial pellet.
