# Supplementary figures and images for: An Influenza HA and M2e Based Vaccine Delivered by a Novel Attenuated Salmonella Mutant Protects Mice against Homologous H1N1 Infection
Source: Front Microbiol. 2017 May 15;8:872. doi: 10.3389/fmicb.2017.00872 (PMC5430049; doi:10.3389/fmicb.2017.00872)

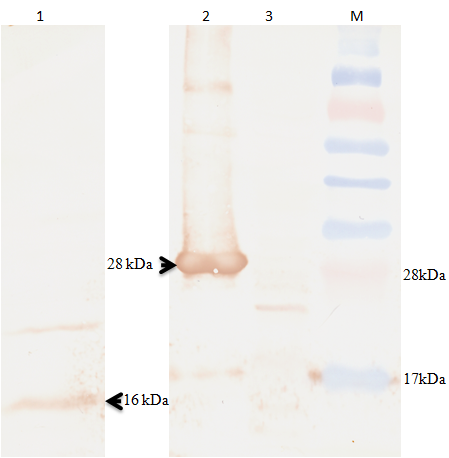

Supplement: FIGURE S1 — Western blot analysis of HA and M2e proteins expressed by JOL1800 strain. The HA1 and multiple tandem copies of M2e proteins expressed by JOL1800 strain were confirmed by Western blot analysis. The JOL1800 bacteria harboring either pJHL65-HA1 plasmid (JOL1917), pJHL65-M2e plasmid (JOL1913) or empty pJHL65 plasmid (JOL1837) were allowed to grow till 0.6 OD600nm. Then bacterial pellets were subsequently subjected to Western blot analysis using poly His-Tag antibody (#AB-TA13002, AprilBio, Co., Ltd, Korea). Lane M, protein Marker (#P8500, GenDEPOT, USA); lane 1, bacterial pellet of JOL1913 showing a 16 kDa band; lane 2, bacterial pellet of JOL1917 showing a 28 kDa band; lane 3, control JOL1837 bacterial pellet. [file Image_1.TIF]
