# Supplementary material for: An Influenza HA and M2e Based Vaccine Delivered by a Novel Attenuated Salmonella Mutant Protects Mice against Homologous H1N1 Infection
Source: Front Microbiol. 2017 May 15;8:872. doi: 10.3389/fmicb.2017.00872 (PMC5430049; doi:10.3389/fmicb.2017.00872)
Supplement: Supplementary file 2 [file Table_1.docx]

**Supplementary Table**

**Table 1 List of bacterial strains, plasmids, and primers used in this study.**

| **Strains / plasmids** | **Description** | **References** | |
| --- | --- | --- | --- |
| DH5α | fhuA2 Δ(argF-lacZ)U169 phoA glnV44 Φ80 Δ(lacZ)M15 gyrA96 recA1 relA1 endA1 thi-1 hsdR17 | Lab stock |  |
| BL21(DE3) | 139(*ara-leu*)7697 *gal*U*gal*K*rps*L (Str^r^)*end*A1 *nup*GF^-^*ompThsdSB*(rB^-^mB^-^)*dcmgalλ*(DE3) pLysSCmr | Lab stock | |
| Χ232 | *E. coli* Δ*asd* strain, used for cloning of genes into *asd*^+^ plasmid | Lab stock | |
| JOL401 | *Salmonella* Typhimurium wild type, SPI-1 *invAE*^+^*hilA*^+^*avr*^+^; SPI-2, amino acid permease^+^; SPI-3, *mgtC*^+^; SPI4, ABC transporter; SPI5, *pipB*^+^; antigen preparation | Lab stock | |
| JOL912  JOL1800 | *∆lon, ∆cpxR, ∆asd* mutant *of S.* Typhimurium  *∆lon, ∆cpxR, ∆asd, ∆wbaP* mutant *of S.* Typhimurium | (Hur and Lee, 2011)  This study | |
| JOL1837  JOL1917 | JOL1800 with pMMP65 empty vector  JOL1800 with pMMP65-HA1 plasmid | Lab stock  This study | |
| JOL1913 | JOL1800 with pMMP65-4M2e plasmid | This study | |
| JOL1912  JOL1925  **Plasmids** | BL21 with pET32a-4M2e plasmid  BL21 with pET28a-HA1 plasmid | This study  This study | |
| pET28a(+) | IPTG-inducible, T7 expression vector, C-terminal 6x His tag, Kan^R^ | Novagen, USA | |
| pET32a(+)  pJHL65 | IPTG-inducible, T7 expression vector, C-terminal 6x His tag, Kan^R^  Asd+, pBR*ori*, lactamase signal sequence based periplasmic secretion plasmid, 6 His-tag, high copy number plasmid | Novagen, USA  (Hur and Lee, 2011) | |
| pJHL65-HA1  pJHL65-M2e  **Primers**  M2e Forward primer 1  M2e Forward primer 2  M2e reverse primer 3 | pJHL65 harboring H1N1 HA1 gene  pJHL65 harboring M2e gene    GCCGAATTCATGAGTCTTCTAACCGAGGT  CGGAGATCTATGAGTCTTCTAACCGAGGT  CGCAAGCTTTCAGGATCCAATAATGTTAGCTGCAACAAC | This study  This study  Designed  Designed  Designed | |
